# FIGO good practice recommendations for vaginal birth after cesarean section

**DOI:** 10.1002/ijgo.70406

**Published:** 2025-07-29

**Authors:** Eytan R. Barnea, Diana Ramasauskaite, Akaninyene Eseme Ubom, Nicoletta Di Simone, Martin Mueller, Anderson Borovac‐Pinheiro, Alice Guarano, Chiara Benedetto, Jolly Beyeza‐Kashesya, Ines Nunes, Bo Jacobsson, Alison Wright, FIGO Childbirth and Postpartum Hemorrhage Committee

**Affiliations:** ^1^ Society for the Investigation of Early Pregnancy New York New York USA; ^2^ Department of Obstetrics, Gynecology and Reproductive Sciences University of Miami Miller School of Medicine Miami Florida USA; ^3^ Center of Obstetrics and Gynecology Vilnius University Medical Faculty Vilnius Lithuania; ^4^ Department of Obstetrics and Gynecology, Faculty of Clinical Sciences PAMO University of Medical Sciences Port Harcourt Nigeria; ^5^ Humanitas University Department of Biomedical Sciences Humanitas University Milan Italy; ^6^ Humanitas San Pio X Milan Italy; ^7^ IRCCS Humanitas Clinical and Research Hospital Milan Italy; ^8^ Department of Obstetrics and Gynecology Lindenhofgruppe Bern Switzerland; ^9^ Department of Pediatrics Maastricht University Medical Centre Maastricht the Netherlands; ^10^ Caism UNICAMP University of Campinas Campinas Brazil; ^11^ Gynaecology and Obstetrics University Division St Anna Hospital Torino Italy; ^12^ FIGO London UK; ^13^ Mulago Specialised Women and Neonatal Hospital Kampala Uganda; ^14^ Department of Obstetrics and Gynecology Gaia/Espinho Local Health Unit Porto Portugal; ^15^ CINTESIS—Center for Health Technology and Services Research University of Porto Porto Portugal; ^16^ Department of Medical Sciences University of Aveiro Aveiro Portugal; ^17^ Department of Obstetrics and Gynecology, Sahlgrenska Academy University of Gothenburg Gothenburg Sweden; ^18^ Department of Obstetrics and Gynecology, Western Health Care Region Sahlgrenska University Hospital Gothenburg Sweden; ^19^ Department of Genetics and Bioinformatics, Division of Health Data and Digitalisation Institute of Public Health Oslo Norway; ^20^ Department of Obstetrics and Gynecology Royal Free London NHS Foundation Trust London UK; ^21^ University College London London UK

**Keywords:** cesarean birth, LMICs, TOLAC, trial of labor after cesarean section, VBAC

## Abstract

The rising global rate of cesarean section (CS) has prompted renewed focus on vaginal birth after cesarean (VBAC) as a safe and effective alternative to repeat CS in properly selected women. The FIGO good practice recommendations provide evidence‐based recommendations to guide VBAC care. Success rates for VBAC range from 60% to 80%, with the highest likelihood in women with prior vaginal birth, non‐recurrent CS indications, interbirth intervals ≥18 months, BMI <30 kg/m^2^, and spontaneous labor onset. While uterine rupture is the primary concern, its risk remains low at approximately 0.3%–0.7% for women with one prior low transverse CS. Comparisons show that VBAC generally results in similar or better maternal and neonatal outcomes than planned repeat CS, including lower maternal mortality, fewer infections, and shorter hospital stays. This article emphasizes appropriate patient counseling, facility readiness for emergency CS, and continuous fetal monitoring during labor. With proper implementation, VBAC offers a valuable strategy to reduce unnecessary repeat surgeries, improve outcomes, and support informed maternal choice.

## 1 INTRODUCTION

Cesarean section (CS) rates are increasing globally and are expected to continue to increase unless intervening measures are taken.^1^, ^2^, ^3^ It is projected that by 2030, nearly 30% of women globally will give birth by CS.^4^ In many countries, primarily in urban areas, primary CS on maternal request, supported by obstetricians, is increasing in frequency.^5^ Primary CS is considered a rapid and safe procedure, with favorable maternal and neonatal outcomes; however, as with any other surgical procedure, CS does carry short‐ and long‐term risks for the mother, baby, and subsequent pregnancies.^1^, ^6^, ^7^ These risks include severe maternal morbidities (hemorrhage requiring hysterectomy or blood transfusion, uterine rupture, anesthetic complications, shock, cardiac arrest, acute renal failure, assisted ventilation, venous thromboembolism, major infection, or in‐hospital wound disruption or hematoma), which are increased three‐fold for CS compared with vaginal birth.^8^ The incidence of placenta accreta spectrum increases with each subsequent CS, from 1% with one prior CS to 3% with three or more prior CSs. After three CSs, the risk that placenta previa will be complicated by placenta accreta is almost 40%.^9^

Vaginal birth after cesarean (VBAC) avoids repeat CS and the associated potential short‐ and long‐term risks. VBAC is generally attempted after one previous CS; however, it may be considered after two prior CSs in carefully selected patients.^10^, ^11^, ^12^ Four key components of VBAC counseling include the individualized risk of uterine rupture, the individualized likelihood of successful VBAC (or risk of intrapartum CS), a patient's future reproductive plans, and patient preference. These components are discussed in these good practice recommendations, which take into account the complexities of VBAC and language sensitivities. The term “trial of labor after cesarean” (TOLAC) has not been used, following feedback from women that this can be perceived as offensive. To provide clarity on the outcomes of VBAC, these are referred to as “successful” and “unsuccessful”.

## 2 RATES OF SUCCESSFUL VBAC

Published data have reported successful vaginal birth rates of 60%–80% after a previous CS.^13^ The rate of successful VBAC is reported as 74.7% in the USA,^14^ 76.6% in Canada,^15^ 80.7% in Taiwan,^16^ 62.8% in Norway,^17^ 61.8% in Nigeria,^18^ 57.6% in the Democratic Republic of Congo,^19^ 69.4% in Addis Ababa, Ethiopia,^20^ and 69.4% in sub‐Saharan Africa.^21^ In counseling women for VBAC, obstetricians should always consider any patient‐specific characteristics that may increase or decrease the likelihood of success above or below the overall rate of 60%–80%; for example, while the success rate of VBAC can be as high as >90% for a woman with one previous CS and a vaginal birth both before and after the CS, the success rate is <60% for women with a recurrent indication for the previous CS, those with body mass index (BMI) >30 kg/m^2^, and those with fetal macrosomia.^13^ The factors affecting the probability of successful VBAC are discussed further in Section 5.

Despite the high overall success rates of VBAC, the attempt rate remains low in many countries. A prospective cohort study in Brazil revealed a primary CS rate of 49.5% that was followed by a second CS in 87.4% of cases.^22^ A large study in the USA, including 4.3 million births, showed that attempted VBAC increased only marginally over a decade, from 15.3% in 2010 to 21.7% in 2020.^14^ Another study of 39 236 775 live births in the USA, recorded between 2010 and 2019, showed that VBAC occurred in only 21.9%, 7.1%, and 4.8% of births with 1, 2, and ≥3 previous CSs, respectively.^10^ In a study of 74 043 singleton term births to women with ≥1 previous CSs in Scotland, only 38.4% of the women had VBAC.^23^ In Norway, a VBAC rate of 70% has been reported among women with cephalic presentation, singleton pregnancies at term.^24^

To increase attempt rates of VBAC, widespread information for women on the risks and benefits of VBAC is important. Furthermore, obstetricians should be trained on how to perform a VBAC safely, with backup systems available on site if patient transfer is required. Patient preference, combined with best available evidence, must be taken into consideration when deciding whether a planned primary CS is clinically indicated. This is important because unnecessary CSs place a major burden on the hospital system, surgical team, and operating room, and require advanced equipment and prolonged postoperative care. Medicolegal frameworks to protect obstetricians and maternity staff from litigation in case of adverse outcomes following VBAC should be instituted, especially as apprehension over litigation remains a significant reason why obstetricians hesitate to offer patients VBAC. Any woman scheduled for VBAC should be adequately counseled on the indication, alternative(s), benefits, and potential risks and complications. Patient understanding of and agreement on the indication, risks, and benefits of VBAC, prior to VBAC performance, may minimize litigations in the event of adverse outcomes.

## 3 RISK OF UTERINE RUPTURE DURING VBAC

The major concern regarding VBAC, and the main reason why clinicians hesitate to offer it, is uterine rupture—a complication that can severely affect both mother and fetus, often requires transfusion, and can lead to an emergency hysterectomy. The risk of uterine rupture differs according to the type of uterine incision that was performed and the number of previous CSs, but overall, the risk is infrequent, with an average rate of 0.3%.^25^ In a study of 17 898 women with a history of CS who underwent VBAC, the overall rate of uterine rupture was 0.7%.^26^ The rates of uterine rupture were 0.7% for women with a prior low uterine segment transverse incision, 2% for those with a prior low vertical incision, 1.9% with a prior classical, inverted T, or J incision, and 0.5% for those with an unknown type of prior incision.^26^ Another study of 18 794 women who birthed at ≥28 weeks of gestation after previous CS reported a uterine rupture rate of 0.5%.^27^ In a systematic review of 14 studies including 4254 women with previous CS, uterine rupture and scar dehiscence occurred in 2.1% of the women.^21^ A meta‐analysis of patients undergoing VBAC after two previous CSs reported a uterine rupture rate of 1.36%.^28^ Macones et al.^29^ reported that uterine rupture occurred in 1.8% of 1082 women with two previous CSs who underwent VBAC. In a retrospective review by Cahill et al.^30^ of 860 women with ≥3 prior CSs, 89 had VBAC and there were no cases of uterine rupture. These studies support that the overall risk of uterine rupture remains low even in women with >1 previous CS. Therefore, more emphasis should be given to identifying women with good potential for successful VBAC.

A limitation of studies reporting the risk of uterine rupture during VBAC is the different definitions of uterine rupture versus uterine dehiscence. While some studies include asymptomatic or minor scar separations, others include only full‐thickness ruptures with clinical consequences. Another limitation is selection bias due to retrospective study design, given that candidates for VBAC are usually carefully selected, with often favorable obstetric factors for VBAC success. Furthermore, most high‐quality data on the risk of uterine rupture during VBAC come from high‐income countries. In low‐ and middle‐income countries (LMICs), access to emergency CS and real‐time fetal monitoring may be limited, which could influence uterine rupture risk and outcomes. Boulvain et al.^21^ and Boatin et al.^31^ reported a uterine rupture rate of 2.1% in women undergoing VBAC in sub‐Saharan Africa, increasing up to 4.9% in Central Africa, compared with 0.3%–0.7% in the USA and other high‐income countries.^25^, ^26^, ^27^ Therefore, data and literature on risk of uterine rupture during VBAC should be interpreted in the context of each study protocol and setting, and these limitations should be borne in mind when counseling women. Aside from type of uterine incision and number of previous CSs, other factors that influence the risk of uterine rupture during VBAC (e.g. gestational age and method of closure of uterine incision at the previous CS, endometritis and wound scar infection complicating the previous CS, prior uterine rupture, interbirth interval) should also be considered during counseling. Some of these are discussed further in Section 5 on the factors affecting the probability of successful VBAC.

## 4 OUTCOMES OF VBAC COMPARED WITH PLANNED REPEAT CESAREAN SECTION

A retrospective cohort study of 3 047 401 women who birthed in Canada between 2003 and 2014 found an increased relative risk but low absolute rate of severe maternal morbidity and mortality with VBAC compared with planned repeat CS (10.7 vs. 5.65 per 1000 births; adjusted RR (aRR) 1.96, 95% CI 1.76–2.19).^32^ Increased relative risk but low absolute risk were also found for neonatal morbidity and mortality (20.8 vs. 14.5 per 1000 births; aRR 1.49, 95% CI 1.38–1.61).^32^ Comparisons between VBAC and planned repeat CS for some specific maternal and neonatal outcomes are discussed in this section. It must be emphasized that randomized controlled trials comparing VBAC with planned repeat CS are lacking due to ethical and feasibility concerns. As a result, the evidence is observational, which limits causal inference and introduces the potential for confounding.

### 4.1 Maternal outcomes

#### Maternal mortality

The National Institute of Child Health and Human Development and the Office of Medical Applications of Research of the National Institutes of Health (NIH) convened a Consensus Development Conference in 2010 to discuss all available evidence related to VBAC. They found that maternal mortality was significantly higher for planned repeat CS than for women who underwent VBAC (13.4/100 000 vs. 3.8/100 000 live births: high grade of evidence).^33^ A literature review of nine studies by Fitzpatrick et al.,^34^ including a median of 7755 women (range, 412–685 137), reported only four maternal deaths, all in women who underwent a planned repeat CS.

#### Uterine rupture

A recent systematic review of 10 studies including 212 440 cases that described uterine rupture demonstrated that uterine rupture was significantly higher in the VBAC group than in the planned repeat CS group (OR 3.35, 95% CI 1.57–7.15; *P* < 0.00001).^35^ Among 47 202 patients in four studies reviewed by Guise et al.,^25^ the risk of uterine rupture in the VBAC and planned repeat CS groups was 0.47% and 0.026%, respectively, with the VBAC group having a significantly higher risk of uterine rupture (RR 20.74, 95% CI 9.77–44.02; *P* < 0.001). On average, uterine rupture occurs in approximately 325/100 000 women undergoing VBAC across all gestational ages, and 778/100 000 women at term. In contrast, the risk of uterine rupture for women undergoing a planned repeat CS is 26/100 000 women when all gestational ages are evaluated, and 22 per 100 000 women at term.^33^

#### Hysterectomy

The overall risk of hysterectomy is statistically similar for VBAC and planned repeat CS (157 vs. 280 per 100 000 women respectively: moderate grade of evidence).^33^ In the study by Fitzpatrick et al.,^34^ the absolute risk of hysterectomy varied from 0% to 0.12% for planned VBAC and 0%–0.61% for planned repeat CS. Twelve of the 14 studies included in the review found no significant difference in the risk of hysterectomy between the VBAC and planned repeat CS groups, while one study found a reduced risk of hysterectomy for VBAC and one reported a higher risk associated with VBAC. Peripartum hysterectomy is most likely performed due to uterine rupture during VBAC and placenta accreta spectrum conditions in planned repeat CS cases.

#### Postpartum hemorrhage

A meta‐analysis of 13 studies by Chen and Mi,^36^ which compared 1892 cases of VBAC with 1703 cases of planned repeat CS, found no statistically significant difference in the incidence of postpartum hemorrhage (PPH) between VBAC and planned repeat CS (2.17% vs. 4.40%; OR 0.83, 95% CI 0.51–1.34, *P* = 0.45).

#### Blood transfusion

The risk of blood transfusion is not significantly different for VBAC or planned repeat CS (900 vs. 1200 per 100 000 women: moderate grade of evidence).^33^ Reported absolute risks of blood transfusion varied from 0% to 3.23% for planned VBAC and 0%–5% for planned repeat CS in the review by Fitzpatrick et al.^34^ Of the 18 studies that investigated the occurrence of blood transfusion in relation to planned VBAC and planned repeat CS, no cases of blood transfusion occurred in one small study, while seven of the larger studies reported an elevated risk of blood transfusion for planned VBAC compared with planned repeat CS (relative effect ranging from 1.14 to 3.73). However, in one of the studies, the elevated risk was only apparent among women without a prior vaginal birth. The remaining 10 studies found no significant difference between planned VBAC and planned repeat CS.^34^

#### Postpartum infection

Reported rates of infection vary widely due to the different definitions and criteria used for infection. Overall, the rates of infection are low (<3% or <3000 per 100 000 women).^33^ Guise et al.^25^ found no significant difference in infection rate between VBAC and planned repeat CS (4.6% vs. 3.2%). Other authors also found no significant differences in rates of infection.^23^, ^36^

#### Deep venous thrombosis

Deep vein thrombosis was assessed in one study and was found to be lower in women undergoing VBAC compared with planned repeat CS (40 vs. 100 per 100 000 women: low grade evidence).^33^

#### Length of hospitalization

Overall, VBAC is associated with shorter hospitalization compared with planned repeat CS.^33^, ^34^

#### Pelvic floor function

No studies on long‐term pelvic floor function have compared women who have VBAC with women who have a planned repeat CS.^29^, ^33^, ^34^, ^36^ Planned repeat CS is theorized to be protective for pelvic floor disorders. However, this is controversial because some of the risk associated with pelvic floor weakness is due to the pregnancy itself and not the method of birth.^37^ While women who have a vaginal birth may have increased risks for pelvic floor disorders (such as stress incontinence or pelvic organ prolapse) compared with women who have a CS, labor progress and the timing of the original CS influence these risks. As such, planned repeat CS for the prevention of pelvic floor disorders should not be considered protective against stress incontinence and prolapse.^33^

### 4.2 Neonatal outcomes

#### Perinatal and neonatal mortality

Studies show that although the absolute risk is low, perinatal and neonatal mortality rates are higher for VBAC than for planned repeat CS (130/100 000 vs. 50/100 000 live births for perinatal mortality and 110/100 000 vs. 50/100 000 for neonatal mortality: moderate grade of evidence).^33^ Fitzpatrick et al.^34^ reported that the absolute risk of perinatal mortality ranged from 0 to 3.8/1000 births for planned VBAC and 0–1/1000 for planned repeat CS, while the absolute risk of neonatal mortality ranged from 0 to 3/1000 for planned VBAC and 0–1/1000 for planned repeat CS.^34^ Landon et al.^26^ found that the frequency of antepartum stillbirth at 37–38 weeks of gestation was higher among women who underwent VBAC than among women who underwent planned repeat CS (0.4% vs. 0.1%; *P =* 0.008). There were no statistically significant differences in rates of antepartum stillbirths at ≥39 weeks of gestation, intrapartum stillbirths, and neonatal mortality.

#### Hypoxic–ischemic encephalopathy

Landon et al.^26^ reported that the frequency of hypoxic–ischemic encephalopathy (HIE) was significantly greater among infants of women who underwent VBAC at term than among the infants of women who had planned repeat CS (12/17 898 vs. 0/15 801; *P* < 0.001). Six of the 12 cases of HIE were associated with induction of labor and oxytocin augmentation. Seven of the 12 cases were associated with uterine rupture. In the cases that occurred without uterine rupture, four women underwent CS due to suspected fetal hypoxia. The NIH systematic review found insufficient data on the incidence of HIE between infants born following VBAC compared with planned repeat CS.^33^

#### Neonatal respiratory morbidity

The absolute risk for transient tachypnea of the newborn among neonates born following VBAC has been reported as 3.6% (95% CI, 0.9–8.0) compared with 4.2% (95% CI, 1.9–7.3) for planned repeat CS, while the absolute risk for neonates needing bag‐and‐mask ventilation for VBAC is estimated at 5.4% (95% CI, 3.5–7.6) and 2.5% (95% CI, 1.6–3.6) for planned repeat CS (low grade of evidence).^25^ There is a lack of data to determine whether substantial differences in respiratory outcomes occur in infants born via planned repeat CS compared with infants born after VBAC.^33^

#### Perinatal asphyxia

Whereas Chen and Mi^36^ reported a statistically significant higher incidence of Apar score <7 at 5 min following VBAC than following planned repeat CS (OR 2.17; 95% CI, 1.69–2.77; *P* < 0.00001), Guise et al.^25^ and Durnwald and Mercer^38^ reported no significant difference in 5‐min Apgar score between VBAC and planned repeat CS.

#### Birth trauma

There is insufficient evidence to draw conclusions on the differences in incidence of birth trauma among neonates born following VBAC and those born following planned repeat CS. Infants born by planned repeat CS are at increased risk of birth trauma, such as fetal lacerations or fetal extraction trauma. Studies of brachial plexus injury show an incidence of 180/100 000 live births among neonates born following VBAC compared with 30/100 000 among neonates born following planned repeat CS. However, there does not appear to be a substantial difference in persistent neurological impairment after brachial plexus injury between VBAC and planned repeat CS.^33^

#### Neonatal intensive care unit admission

Chen and Mi^36^ reported that there was no statistically significant difference in the rate of neonatal care unit (NICU) admission between VBAC and planned repeat CS (OR 1.06; 95% CI, 0.96–1.16; *P* = 0.27). In the review by Guise et al.,^25^ five of the six studies that measured NICU admission found no significant differences in frequency of NICU admissions between VBAC compared with planned repeat CS. One study reported a significant increase in NICU admissions of neonates born after VBAC compared with planned repeat CS.

#### Neonatal sepsis

There is insufficient/low‐grade evidence on neonatal sepsis following VBAC compared with planned repeat CS to make any meaningful conclusions.^25^, ^33^

## 5 FACTORS AFFECTING THE PROBABILITY OF SUCCESSFUL VBAC

Factors affecting the probability of successful VBAC can be divided into prognostic factors for VBAC success (Table 1) and factors affecting the risk of uterine rupture during VBAC (Table 2).

**TABLE 1:** Prognostic factors for success of vaginal birth after cesarean section.

Factors	Odds of VBAC success (95% CI) (absolute VBAC success rate)
Previous VBAC (vs. no previous VBAC)	5.3 (2.20–12.69) (94.0% vs. 65.0%) [39, 40]
Previous SVB (vs. no previous SVB)	4.2 (3.90–4.60) (86.6% vs. 60.9%) [41]
Non‐recurrent indication for previous CS (breech vs. CPD)	1.7 (1.38–2.01) (88.6% vs. 63.8%) [42, 43]
Maternal BMI <30 kg/m^2^ (vs. ≥30 kg/m^2^)	1.8 (1.11–2.97) (79.6% vs. 68.4%) [41, 44]
Younger maternal age <35 years (vs. ≥35 years)	1.5 (1.00–2.36) (73.6% vs. 59.6%) [41, 44, 45]
Estimated fetal weight <4000 g (vs. ≥4000 g)	2.1 (1.80–2.40) (74.9% vs. 62.0%) [41]
Gestational age 37–41 weeks (vs. >41 weeks)	2.8 (1.10–7.10) (84.2% vs. 65.7%) [46]
Spontaneous labor (vs. induced labor)	3.1 (1.52–6.17) (84.0% vs. 65.0%) [19, 40]
Bishop cervical score of ≥6 (vs. <6) or cervical dilatation of ≥4 cm (vs. <4 cm) at admission in labor	3.8 (2.17–6.53) (83.8% vs. 66.8%) [41, 42]

Abbreviations: BMI, body mass index; CPD, cephalopelvic disproportion; CS, cesarean section; SVB, spontaneous vaginal birth; VBAC, vaginal birth after cesarean.

**TABLE 2:** Risk factors for uterine rupture during vaginal birth after cesarean section.

Factors	Odds of uterine rupture (95% CI) (absolute uterine rupture rate)
Number of previous cesarean sections (2 vs. 1)	2.0 (1.24–3.27) (1.8% vs. 0.9%) [29]
Previous low segment uterine incision cesarean section	Only option [47, 48, 49, 50]
Type of previous low segment uterine incision (low vertical vs. low transverse)	1.0 (0.29–3.45) (0.8% vs. 1.0%) [48]
Single versus double layer closure of previous low uterine incision	1.9 (0.63–5.62) (0.7% vs. 0.3%) [51]
Interbirth interval <18 months (vs. ≥18 months)	3.0 (1.30–7.20) (4.8% vs. 1.9%) [52]
Induced versus spontaneous labor	2.9 (1.75–4.67) (1.0% vs. 0.4%) [26]
Augmented versus non‐augmented labor	2.4 (1.49–3.93) (0.9% vs. 0.4%) [26]

### 5.1 Prognostic factors for VBAC success

#### Previous vaginal birth and VBAC

A previous vaginal birth, especially a prior VBAC, has been consistently associated with successful VBAC. A recent systematic review and meta‐analysis by Mekonnen and Asfaw^39^ indicated that the odds of successful VBAC were three times higher among women with a history of previous spontaneous vaginal birth (adjusted OR (aOR) 2.92; 95% CI, 2.02–4.23) compared with women who had no past spontaneous vaginal birth. They also documented that women with a previous VBAC had five times higher odds of having a successful VBAC (aOR 5.29; 95% CI, 2.20–12.69) compared to their counterparts without a previous VBAC.^39^ Similarly, this has been reported by other systematic reviews and meta‐analyses by Wu et al.^42^ and Eden et al.^53^ In their systematic review, Eden et al.^53^ reported that women with a previous VBAC were significantly more likely to have another VBAC compared with women with a previous vaginal birth that occurred before the prior CS (combined OR 4.39; 95% CI, 2.87–6.72 vs. combined OR 1.60; 95% CI, 1.22–2.09).

In their retrospective cohort study of 9960 women who gave birth in 16 community and university hospitals, Elkousy et al.^40^ reported a VBAC rate of 65% for women with no history of vaginal birth, 83% for women with a previous vaginal birth before CS, 94% for women with a previous VBAC, and 93% for women with a vaginal birth both before and after the previous CS. A secondary analysis of the National Institute of Child Health and Human Development Maternal‐Fetal Medicine Units Network cohort data of 13 532 women reported that VBAC success increased with the number of prior VBACs.^54^ Women with 0, 1, 2, 3, and 4 prior VBACs had VBAC rates of 63.3%, 87.6%, 90.9%, 90.6%, and 91.6%, respectively (*P* < 0.001).^54^

#### Non‐recurrent indication for previous cesarean section

Women whose previous CS was for non‐recurring indications such as breech presentation or non‐reassuring fetal status have higher chances of VBAC success than for indications that can recur, such as an arrest of labor disorder.^13^, ^33^, ^55^ Eden et al.^53^ reported in their systematic review that women with a previous CS indication of insufficient labor progress were less likely to have a successful VBAC (combined OR 0.53; 95% CI, 0.44–0.63) than women with other CS indications. Similarly, in a more recent systematic review and meta‐analysis, Wu et al.^42^ showed that fetal malpresentation as the indication for previous CS significantly increased the likelihood of VBAC success (OR 1.66; 95% CI, 1.38–2.01).

One retrospective study showed that a previous CS due to malpresentation significantly increased the likelihood of successful VBAC (aOR 7.4; 95% CI, 2.8–19.2) compared with an indication for insufficient labor progress.^46^ Another retrospective study, by Weinstein et al.,^43^ further supported this finding and reported that malpresentation (primarily breech) as the indication for the primary CS was significantly associated with successful VBAC (OR 1.9; 95% CI, 1.0–3.6; *P =* 0.05) compared with a non‐breech indication. Weinstein et al.^43^ documented a VBAC success rate of 88.6% when breech presentation was the indication for the previous CS compared with 63.8% when the indication was cephalopelvic disproportion/insufficient labor progress.

#### Estimated fetal weight <4000–4500 g

Fetal macrosomia (defined as fetal weight >4000 g) has been associated with a higher likelihood of unsuccessful VBAC.^13^, ^15^ Flamm and Goings^56^ conducted an analysis of the outcomes of 301 VBACs with birth weights ≥4000 g. Whereas 58% (139/240) birthed vaginally in the 4000–4499 g birthweight group, 43% (26/61) birthed vaginally in the >4500 g birthweight group. When compared with 1475 VBACs with birth weights <4000 g, no significant differences in perinatal or maternal morbidity were found. Comparison with a control group of 301 women with no previous CS who birthed macrosomic neonates also demonstrated no significant differences in perinatal or maternal morbidity.

A recent multicenter French study retrospectively reviewed 235 women with a single prior CS, who were carrying a singleton pregnancy with suspected fetal macrosomia (estimated fetal weight <4500 g), and who had attempted VBAC (*n* = 170) or planned repeat CS (*n* = 65) at ≥37 weeks' gestational age.^57^ Of the 170 women who attempted VBAC, VBAC was successful in 68.8%. There were no significant differences between the VBAC and planned repeat CS groups in the rates of uterine rupture, PPH, blood transfusion, Apgar scores, neonatal hospitalization, and fetal trauma. Estimated fetal weight >4000 g was more frequent in the planned repeat CS group (*P* = 0.011) but there was no significant difference in fetal birth weight between the two groups (planned repeat CS: 3865 g (3656–4168 g) vs. VBAC: 3815 g (3597–4085 g; *P* = 0.068)).

Another retrospective cohort study of 276 primiparous women with macrosomic fetuses weighing ≥4000 g, who attempted VBAC after CS at their first birth, found a VBAC success rate of 63%.^58^ Spontaneous onset of labor (aOR 3.68; 95% CI, 2.05–6.61; *P* < 0.001), epidural anesthesia (aOR 2.38; 95% CI, 1.35–4.20; *P* = 0.003), and history of CS due to non‐arrest disorder (aOR 2.25; 95% CI, 1.32–3.85; *P* = 0.003) were significantly associated with VBAC success. VBAC was successful in 82% of cases when all three favorable factors were present, 61.3% of cases in the presence of two favorable factors, and 38.6% of cases when ≤1 of these three factors was present (*P* < 0.001).

Evidence on the incidence of uterine rupture during VBAC with fetal macrosomia is conflicting.^40^, ^56^, ^57^, ^59^, ^60^ While Elkousy et al.^40^ reported a uterine rupture rate of 2.8% with estimated fetal weight of ≥4000 g compared to 1.2% with fetal weight <4000 g (RR 2.3; *P <* 0.001), Chamagne et al.^57^ recorded no case of uterine rupture in 170 women with fetal macrosomia who underwent VBAC. Leung et al.^60^ also found no relationship between fetal macrosomia and uterine rupture. From available evidence, while VBAC can be offered up to an estimated fetal weight of 4500 g, fetal weight of >4000 g increases the likelihood of unsuccessful VBAC.^15^, ^55^, ^61^, ^62^

#### Maternal body mass index <30 kg/m^2^

In a recent population‐based cross‐sectional study, Jude et al.^63^ compared 126 809 women with obesity who underwent VBAC with 667 469 women who had a planned repeat CS at term. The overall composite adverse maternal outcomes were significantly higher for patients with obesity undergoing VBAC compared with planned repeat CS (9/1000 live births vs. 5.3/1000 live births; aRR 1.64; 95% CI, 1.53–1.75). Similarly, Yao et al.^64^ compared outcomes between VBAC and planned repeat CS in a retrospective cohort analysis of 538 264 pregnancies with singleton term births, which were complicated by maternal obesity (BMI ≥30 kg/m^2^) and history of one or two previous CSs. They concluded that women with obesity who underwent VBAC had increased risks of maternal (blood transfusion, uterine rupture, and ICU admission) and neonatal (low 5‐min Apgar score, NICU admission, neonatal seizure, and neonatal death) complications compared with planned repeat CS. Juhasz et al.^65^ evaluated VBAC success rates in 1213 women and stratified these by BMI. The VBAC success rates for BMI <19.8, 19.8–26, 26.1–29, and >29 kg/m^2^ were 83.1%, 79.9%, 69.3%, and 68.2%, respectively (*P* < 0.001).

#### Favorable labor characteristics for VBAC

Spontaneous onset of labor, vertex presentation, cervical dilatation ≥4 cm or Bishop score ≥6 at presentation in labor, fetal head at station 0 or lower, epidural analgesia, and gestational age of 37–41 weeks are associated with a higher likelihood of VBAC success.^15^, ^19^, ^41^, ^66^, ^67^, ^68^

### 5.2 Factors affecting risk of uterine rupture during VBAC

#### Number of previous cesarean sections

Whereas a history of two previous CSs is often regarded as an absolute contraindication to VBAC, more recent evidence suggests that women—in highly selected cases—with two prior CSs may be considered for an attempt at VBAC if there is appropriate emergency obstetric care immediately available, in a high‐resource setting, and where the woman herself has a strong conviction and is well‐informed about the associated risks.

A retrospective cohort study compared outcomes between 82 women with two prior CSs who attempted VBAC (including those with a previous vaginal birth) and 711 women with two prior CSs who underwent planned repeat CS.^69^ In the attempted VBAC group, 69.5% achieved a successful vaginal birth. In comparison to those who had an unsuccessful VBAC, women who had a successful VBAC had a lower mean BMI at admission (31.0 kg/m^2^ vs. 34.5 kg/m^2^; *P* = 0.04), lower mean neonatal birth weight (3351 g vs. 3681 g; *P* = 0.01), and a higher likelihood of a previous vaginal birth and a previous successful VBAC. Induction or augmentation of labor was associated with a greater likelihood of unsuccessful VBAC. There was one case of uterine rupture in the VBAC group (1/82) and none in the planned repeat CS group (0/711). However, there was one unplanned hysterectomy in the planned repeat CS group. Maternal and neonatal morbidity and mortality were not significantly different between the groups.^69^

Tahseen and Griffiths^28^ conducted a systematic review and meta‐analysis of 17 studies of 5666 women undergoing labor after one or two CSs to compare success rates and adverse outcomes after two CSs (VBAC‐2) versus after one CS (VBAC‐1) and planned repeat third CS. The success rate of VBAC‐2 was 71.1%, with a uterine rupture rate of 1.36%. VBAC‐2 had a significantly lower success rate compared with VBAC‐1 (71.1% vs. 76.5%; *P* < 0.001) and higher rates of uterine rupture (1.59% vs. 0.72%; *P* < 0.001) and hysterectomy (0.56% vs. 0.19%; *P* = 0.001). Nonetheless, maternal morbidity in the VBAC‐2 group was comparable to that in the planned repeat CS group. No significant differences were found in neonatal morbidity or mortality across the VBAC‐1, VBAC‐2, and planned repeat CS groups.

Macones et al.^29^ compared 12 535 women with one previous CS with 1082 women with two prior CSs who attempted VBAC. The VBAC success rates were similar between the two groups (75.5% vs. 74.6%; *P* = 0.50). However, the odds of major maternal morbidity were higher in the group with two prior CSs (aOR 1.61; 95% CI, 1.11–2.33). Compared with planned repeat CS, women with two prior CSs who underwent VBAC had low absolute risk but an increased risk of major complications (aOR 2.26; 95% CI, 1.17–4.37).

Limited data suggest that VBAC after three prior CSs may have similar success rates to those after one prior CS. In a study by Cahill et al.,^31^ 860 women with three prior CSs were evaluated, of whom 89 attempted VBAC. The VBAC success rate was comparable to that of women with one prior CS (79.8% vs. 75.5%; aOR 1.4; 95% CI, 0.81–2.41; *P* = 0.22), with no case of composite maternal morbidity reported.

#### Type of uterine incision at previous cesarean section

Naef et al.^47^ evaluated 322 women with a previous low‐segment vertical CS, of which 174 attempted VBAC and 148 had planned repeat CS. VBAC success rate was 83% among those who attempted VBAC. Uterine rupture occurred in two patients (1.1%) in the VBAC group and none in the planned repeat CS group. The incidence of PPH was similar between the two groups but endometritis developed significantly more in the planned repeat CS group. Maternal and neonatal morbidity and mortality were similar between the two groups.

Shipp et al.^48^ compared the outcomes of 2912 women with a prior low uterine transverse incision and 377 women with previous low vertical incision undergoing VBAC. Women whose low vertical incision extended into the corpus of the uterus were excluded. The scar disruption (1.3% for the low transverse group vs. 1.6% in the low vertical group; *P =* 0.6) and symptomatic rupture rates (1% in the low transverse group vs. 0.8% in the low vertical group; *P* > 0.999) were similar for the two groups.

Landon et al.^26^ reported a uterine rupture rate of 0.7% for women with a prior low transverse incision undergoing VBAC compared with 2% for those with a prior low vertical incision. The risk of uterine rupture may be as high as 4%–9% in women with incisions that extend into the contractile portion of the uterus (upper uterine segment).^49^ Greene et al.^50^ reviewed 62 CSs involving a vertical upper uterine segment incision (including five inverted‐T incisions). In 15 subsequent pregnancies, scar rupture and scar dehiscence rates were 6.2% and 12.5%, respectively.

#### Unlocked single‐ or double‐layer closure of low uterine incision at previous cesarean section

A systematic review and meta‐analysis by Roberge et al.^70^ reported no significant difference between single‐ versus double‐layer closure for uterine scar dehiscence or uterine rupture. Despite lower residual myometrial thickness noted in women with single‐layer closure compared with those with double‐layer closure, there was no significant difference in the risk of uterine scar defect with single‐layer closure compared with double‐layer closure. A continuous, locked, single‐layer closure was coupled with a larger scar defect, which was also reported by Qayum et al.^51^ However, Bennich et al.^71^ reported that double‐layer closure did not increase residual myometrial thickness compared with single‐layer closure when an unlocked technique was used. More recent systematic reviews and meta‐analyses by Di Spiezio Sardo et al.^72^ and Qayum et al.^51^ have similarly shown that single‐ and double‐layer closure of the uterine incision following CS are associated with a similar incidence of cesarean scar defect, as well as uterine dehiscence and rupture in a subsequent pregnancy. Based on this evidence, we recommend that either a one‐ or a two‐layer closure technique is used; if a single‐layer closure is performed, a continuous unlocked technique should be used.^73^

#### Interbirth interval

In their multicenter retrospective cohort study to determine the optimal interbirth interval for VBAC, Rao et al.^74^ evaluated 1080 pregnant women with one or two CSs who attempted VBAC. They found that an interbirth interval of <24 months did not show a statistically significant association with uterine rupture, but was significantly associated with PPH, preterm birth, and low birth weight compared with women with an interbirth interval of 24–59 months.

A secondary analysis of a retrospective cohort study of 1768 women who underwent VBAC reported that after adjustment for confounders, an interbirth interval of <18 months was significantly associated with an increased risk of uterine rupture (OR 3.0; 95% CI, 1.3–7.2), whereas an interbirth interval of 18–24 months was not (OR 1.1; 95% CI, 0.4–3.2).^52^ Similarly, Shipp et al.^75^ reported that women with an interbirth interval of <18 months had three times the odds of uterine rupture compared with women who had an interbirth interval of >18 months. In a cohort of 1185 women who attempted VBAC, the success rate for women with an interbirth interval of <19 months was comparable with the success rate for women with an interbirth interval of ≥19 months (79.0% vs. 85.5%; *P =* 0.12).^76^ However, for women who had induction of labor, an interbirth interval of <19 months was significantly associated with reduced VBAC success rate when compared with an interbirth interval of ≥19 months (14.3% vs. 86.1%; *P* < 0.01).

The study by Kessous and Sheiner,^77^ which included 3176 patients, evaluated the safety of women undergoing VBAC who had a short interbirth interval. The study concluded that a short interbirth interval (<12 months) is not a risk factor for major complications such as uterine rupture, perinatal death, and maternal death, but that it is a risk factor for preterm birth. Esposito et al.^78^ and Stamilio et al.^79^ reported that an interpregnancy interval of <6 months is associated with increased risks of uterine rupture, major morbidity, and blood transfusion. The study by Stamilio et al.^79^ was a secondary analysis of a multicenter, retrospective cohort study in the USA of 13 331 pregnant women.

#### Measurement of lower uterine segment thickness to predict uterine rupture during VBAC

Sonographic measurement of lower uterine segment thickness has been used antenatally in women with a previous CS to predict uterine rupture during VBAC. Kok et al.^80^ conducted a meta‐analysis of 21 studies that included 2776 patients to evaluate the accuracy of antenatal sonographic measurement of lower segment thickness for the prediction of risk of uterine rupture during VBAC. They reported that a full‐thickness cutoff of 3.1–5.1 mm and a myometrial thickness cutoff of 2.1–4.0 mm provided a strong negative predictive value for the occurrence of a uterine defect during VBAC. A myometrial thickness cutoff of between 0.6 and 2.0 mm provided a strong positive predictive value for the occurrence of a defect, with sensitivity and specificity of 76% and 92%, respectively. Kement et al.^81^ reported that 82.6% of patients with dehiscence had full lower uterine segment thickness of 1–2 mm and 95.7% had full thickness of <1 mm. A large multicenter prospective study, which included 984 women undergoing VBAC, reported that uterine scar dehiscence occurred in 5.3% of the women with a lower uterine segment thickness of 2–2.4 mm compared with 1.9% in women with a lower uterine segment thickness of ≥2.5 mm.^82^

These studies provide support for the use of antenatal measurements of lower uterine segment thickness for the prediction of uterine rupture in women undergoing VBAC; however, no ideal thickness cutoff value has been defined for clinical use. Furthermore, the technical aspects of the measurements are not standardized in terms of full thickness versus myometrial thickness measurements, use of transabdominal versus transvaginal ultrasound for measurements, and measurements with a full versus empty bladder. The clinical applicability of lower uterine segment thickness measurement in counseling patients whether to attempt VBAC needs to be further assessed in prospective observational studies, using a standardized method of measurement.

## 6 SPECIAL CONSIDERATIONS AND SITUATIONS

### 6.1 Multiple pregnancy and VBAC

Shinar et al.^83^ performed a systematic review of VBAC success rates and maternal and neonatal outcomes in twin pregnancy versus planned repeat CS. They evaluated 2336 VBAC cases and 5763 planned repeat CS cases. Rates of successful VBAC and of uterine rupture were 72.2% (95% CI, 59.7–83.2) and 0.87% (95% CI, 0.51–1.31), respectively. VBAC was associated with a significantly higher risk of neonatal death (which the authors theorized may have been influenced by preterm birth), with no significant differences in mean gestational age at birth, NICU admission, or Apgar score <7 at 5 min. The risk of maternal infectious morbidity was significantly lower with VBAC, while the risks of uterine scar dehiscence, blood transfusions, and hysterectomy were comparable.

A systematic review and meta‐analysis by Kabiri et al.^84^ included 8209 twin pregnancies with previous CS, of which 2484 underwent VBAC and 5725 had a planned repeat CS. Although the rate of uterine rupture was higher in the VBAC group compared with the planned repeat CS group, the rates of uterine rupture and successful VBAC were similar for women with twins and those with singleton pregnancies. Women who attempted VBAC with twins did not have increased risks of uterine scar dehiscence, hemorrhage, blood transfusion, or neonatal morbidity and mortality compared with women who had planned repeat CS.

### 6.2 Breech presentation and external cephalic version during VBAC

Paul et al.^85^ conducted a prospective cohort study to evaluate maternal and neonatal outcomes following vaginal breech birth at term after CS. They evaluated 604 women who had a vaginal breech birth, of which 567 were primiparous and 37 had a history of a prior CS. The vaginal birth rate of the women with a prior CS was 51.4% compared with 60.7% in the primiparous group. There were no significant differences in maternal and neonatal outcomes between the two groups. CS rate among women with a prior CS was 49%, which was not significantly higher than the CS rate of 39% in the primiparous group. Current guidelines agree that breech presentation is not an absolute contraindication to VBAC.^15^, ^63^

External cephalic version (ECV) is not contraindicated in women with a previous CS.^15^, ^55^, ^61^ The likelihood of successful ECV has been reported to be similar in women with or without prior CS. A systematic review and meta‐analysis of eight studies totaling 14 515 women who underwent ECV, comprising 1215 with a previous CS and 13 300 without a previous CS, reported a median ECV success rate of 74% (IQR 63%–81%) in women with a previous CS, which was similar to a success rate of 69% (IQR 64%–83%) in women without a previous CS (pooled OR 0.84; 95% CI, 0.61–1.15).^86^ However, the overall success rate of subsequent vaginal birth in women with a previous CS was lower at a median point prevalence of 75% (IQR 61%–84%) compared with 92% (IQR 85%–95%) in women without a previous CS (pooled OR 0.26; 95% CI, 0.14–0.50). Another systematic review by Zhang and Ward^87^ evaluating the safety and efficacy of ECV after a previous CS included nine studies involving 1264 women. The ECV success rate ranged from 50% to 100%, with subsequent vaginal birth rates of 50%–74.9%. There was no case of uterine rupture. Both systematic reviews and meta‐analysis concluded that ECV can be performed safely in women with a previous CS, with similar success rates when compared with women without a previous CS.

### 6.3 Induction and augmentation of labor during VBAC

A meta‐analysis of 14 cross‐sectional studies that included 48 457 women who attempted VBAC showed a significantly higher rate of VBAC success in the spontaneous labor group compared with the induced labor group (74.3% vs. 60.7%; *P =* 0.001) and a significantly lower rate of uterine rupture (0.7% vs. 2.2%; *P =* 0.0003).^88^ The rate of uterine rupture in women using oxytocin in VBAC was significantly higher than in women not using oxytocin (1.4% vs. 0.5%; *P* = 0.0002). In addition, the rate of uterine rupture following oxytocin augmentation among women with spontaneous labor was lower than in women who were induced, although the difference was not significant (1.7% vs. 2.2%; *P* = 0.443).

Observational studies have reported rates of uterine rupture of 0.4%–0.52% for spontaneous labor, 0.77%–0.9% for labor induced without prostaglandins, 0%–2.45% for labor induced with prostaglandins alone, 1.4% for labor induced with prostaglandins with or without oxytocin, 1.1% for labor induced with oxytocin alone, and 0.9% for augmented labor.^26^, ^89^ A Cochrane review that evaluated methods of labor induction for women with a previous CS concluded that there was insufficient evidence and the studies were underpowered to determine the optimal method of labor induction in women with a prior CS.^90^

Based on available evidence, current guidelines recommend the use of mechanical methods, such as amniotomy and transcervical extra‐amniotic Foley catheter, over prostaglandins for cervical ripening and induction of labor in women with a previous CS.^15^, ^55^, ^68^, ^91^ Misoprostol (prostaglandin E_1_) should not be used for cervical ripening and induction of labor because it is associated with an increased risk of uterine rupture in women with a previous CS.^15^, ^55^, ^92^ Some studies have suggested that low dose dinoprostone (prostaglandin E_2_) is a safe option for induction of labor in women undergoing VBAC, with no appreciable increase in rates of uterine rupture or maternal and perinatal mortality when compared with women undergoing a spontaneous VBAC.^93^, ^94^

Compared with induction of labor at >41 weeks, induction at 39–41 weeks is associated with lower odds of CS and greater odds of VBAC success.^55^, ^95^, ^96^ Gestational age ≥41 weeks increases the odds of uterine rupture.^15^, ^27^

### 6.4 Assisted vaginal birth during VBAC

There is evidence to suggest that in carefully selected women undergoing VBAC, a trial of low forceps or outlet forceps or vacuum may be safely attempted by a skilled obstetrician in an operating theater, where immediate recourse to CS can be undertaken, in case of any complication.^97^, ^98^ This is discussed in detail, with recommendations for practice, in an upcoming paper on assisted vaginal birth from the FIGO Committee on Childbirth and PPH.

## 7 REQUIREMENTS FOR VBAC

These include antenatal assessment and counseling, infrastructure and setting, and intrapartum management.

### 7.1 Antenatal assessment and counseling

Review of the operative notes from the previous CS is important so that clinicians are aware of the indication for the CS, location, type, and method of closure of the uterine incision.^99^ The obstetric history and antenatal records of the patient should also be reviewed to exclude any contraindications to VBAC and vaginal birth. The patient should be thoroughly counseled on the risks and benefits of VBAC versus planned repeat CS. The chance of a successful VBAC and the risk of uterine rupture should be evaluated before deciding whether to attempt VBAC.^99^ Several prediction models for VBAC success exist but many lack external validation and are at high risk of bias, hindering generalizability and applicability.^100^

### 7.2 Required infrastructure and setting

VBAC should be performed in facilities that have resources for 24‐h emergency CS, blood bank, and NICU backup owing to the associated maternal and neonatal risks.^101^

On admission in labor, supportive one‐to‐one nursing/midwifery care and continuous electronic fetal monitoring is the standard, given that fetal heart rate abnormality is the most common sign of uterine rupture—seen in up to 70% of cases.^15^, ^55^ Other clinical features of uterine rupture include severe abdominal pain, especially if persisting between uterine contractions, acute scar tenderness, vaginal bleeding, hematuria, cessation of previously adequate uterine contractions, loss of station of the fetal presenting part, prominent/easily palpable fetal parts through the abdomen, maternal tachycardia, hypotension, and fainting or shock.^68^

Epidural analgesia is not contraindicated but an increasing demand for pain relief should elicit a suspicion of uterine rupture.^68^ Epidural analgesia does not appear to mask the signs and symptoms of uterine rupture,^55^, ^102^, ^103^ and fetal heart rate abnormalities remain the first and most common sign of uterine rupture. Furthermore, epidural analgesia can provide more rapid unplanned intrapartum CS of a compromised fetus than induction of general anesthesia.^61^ A multicenter prospective cohort study on the effect of epidural analgesia on maternal and neonatal outcomes during VBAC included 423 multiparous women underdoing VBAC, out of whom 263 women received epidural analgesia during labor and 160 did not. The success rate of VBAC was significantly higher in women who received epidural analgesia compared with those who did not (85.6% vs. 69.4%; *P* < 0.01), with no increased risks of PPH, uterine rupture, or adverse neonatal outcomes.^104^ A more recent and larger retrospective population‐based cohort study including 17 516 women with a previous CS, of whom 2652 used epidural analgesia during labor and 14 864 did not, also found similar neonatal outcomes for both groups of women.^105^

### 7.3 Intrapartum management

Labor progress should be assessed by the same standards as in women with an unscarred uterus as the available evidence shows that women attempting VBAC have similar labor patterns as women who have not had a prior CS.^106^, ^107^ Management of the second and third stages of labor is not different in women undergoing VBAC but there should be a low threshold for assisted vaginal birth or emergency intrapartum CS if uterine rupture is suspected. In case of a retained placenta, abnormal placentation should be considered. Manual removal in such situations should be attempted in the operating room if placenta accreta spectrum disorders are confirmed. Bedside ultrasound can be used to evaluate the placentation before manual removal.

Routine uterine exploration after VBAC is not recommended in a hemodynamically stable patient with no abnormal vaginal bleeding.^55^, ^61^ However, excessive vaginal bleeding or signs of hypovolemia may indicate uterine rupture and should prompt a complete evaluation of the genital tract. If the uterus is explored and a dehiscence is diagnosed, and the patient is stable without abnormal vaginal bleeding, blood analysis should be performed to exclude intraperitoneal bleeding, which would be an indication for laparotomy and repair if confirmed. Surgical repair of asymptomatic uterine scar dehiscence suspected by uterine exploration after VBAC is not recommended, as it has not been shown to improve outcomes.^55^, ^61^

## 8 SUMMARY OF FIGO RECOMMENDATIONS FOR VBAC

- **Fetal macrosomia**
  1. Fetal macrosomia alone is not an absolute contraindication to VBAC, but women with macrosomic fetuses planning VBAC should be counseled regarding the higher likelihood of failure and possible increased risk of uterine rupture.
  2. While VBAC can potentially be offered up to an estimated fetal weight of 4500 g, a fetal weight of ≥4000 g increases the likelihood of unsuccessful VBAC.
  3. Planned repeat CS should be offered when estimated fetal weight is >4500 g.
  4. Favorable factors that increase the likelihood of success in women with suspected fetal macrosomia undergoing VBAC include one previous low transverse CS for a non‐recurrent indication, a prior vaginal birth and prior VBAC, singleton pregnancy, cephalic presentation, term gestation, and spontaneous onset of labor.
- **Number of previous cesarean sections**
  5 Women with a single previous CS may be offered VBAC in the absence of any contraindication to vaginal birth. This should be accompanied by appropriate counseling and close intrapartum monitoring.
  6 VBAC is generally not recommended after two previous CSs. However, if a woman with two prior CSs and a vaginal birth strongly desires a VBAC, the patient should receive thorough counseling from a senior obstetrician on the associated risks and potential complications, including the increased likelihood of VBAC failure, cesarean birth, and uterine rupture. If the patient still wishes to consider VBAC, this option may be available in some high‐resource facilities. These facilities must have the following prerequisites:
    - one‐to‐one nursing/midwifery care.
    - continuous fetal monitoring.
    - on‐site availability of blood products and transfusion services.
    - immediate access to surgical theater facilities.
    - presence of a surgeon capable of performing emergency hysterectomy on site.
  7 There are limited data on the risks of uterine rupture and overall safety to recommend VBAC in women with three or more previous CSs. Therefore, a planned repeat CS is recommended at three or more previous CSs.
- **Type of uterine incision for previous cesarean section**
  8 VBAC is safe and associated with low absolute risks of uterine rupture in women with a previous low transverse or low vertical uterine incision (usually described as a vertical incision on the lower (non‐contractile) uterine segment).
  9 VBAC is contraindicated in women with previous classical CS (a vertical incision on the upper (contractile) uterine segment), inverted T or J incisions, and previous incisions that extend into the upper uterine segment. A planned repeat CS is recommended in these cases due to the higher risk of uterine rupture.
- **Method of closure of uterine incision at previous cesarean section**
  10 Single‐layer closure of a previous low‐segment CS is not an absolute contraindication to VBAC.
- **Interbirth interval**
  11 An interbirth interval of at least 18 months is recommended for VBAC for optimal maternal and neonatal outcomes.
  12 Women with an interbirth interval of <18 months who are attempting VBAC should be counseled on the higher likelihood of unsuccessful VBAC and adverse outcomes.
- **Prediction of uterine rupture and VBAC success**
  13 Routine use of antenatal lower uterine segment thickness measurements for the prediction of uterine rupture in women undergoing VBAC is not recommended, as no ideal thickness cutoff value has been defined for clinical use. Clinical applicability of lower uterine segment thickness measurement in counseling patients whether to attempt VBAC needs to be assessed in prospective observational studies using a standardized method of measurement.
  14 Routine use of prediction models to predict VBAC success is not recommended as many of the models lack external validation and are at high risk of bias, hindering generalizability and applicability.
- **Multiple pregnancy**
  15 Twin pregnancy is not an absolute contraindication to VBAC.
  16 VBAC can be a safe alternative to a planned repeat CS in women with twin gestation and a history of one previous CS with a low transverse incision in the presence of other favorable factors for VBAC, with careful counseling and monitoring.
  17 A planned repeat CS is recommended for women with higher order multiple pregnancy with a previous CS.
- **Breech presentation and external cephalic version**
  18 Breech presentation is not an absolute contraindication to VBAC in women with a single fetus in breech presentation and a history of one previous low transverse CS, who are otherwise good candidates for VBAC.
  19 External cephalic version can be safely attempted in women with a singleton breech presenting fetus and a prior low transverse uterine incision, who are good candidates for VBAC and external cephalic version.
- **Induction and augmentation of labor during VBAC**
  20 Induction of labor is not contraindicated in women with one previous low uterine incision, who are otherwise good candidates for VBAC.
  21 Women should be well informed that induction of labor is associated with a lower VBAC success, as well as increased risk of uterine rupture.
  22 When indicated in women with a previous CS, induction of labor should be performed between 39 and 41 weeks of gestation to increase the chances of success and minimize the risk of uterine rupture.
  23 Mechanical methods such as amniotomy and transcervical Foley catheter are recommended for cervical ripening and induction of labor in women with a previous CS.
  24 Misoprostol (prostaglandin *E*_1_) should not be used for cervical ripening and induction of labor in women with a previous CS because it is associated with a high risk of uterine rupture. Prostaglandins can be considered if labor induction is indicated in the second trimester.
  25 Use of oxytocin for induction of labor or augmentation of labor is not contraindicated in women undergoing VBAC. It should, however, be used cautiously and women should be counseled on the increased risk of uterine rupture.
- **Assisted vaginal birth during VBAC**
  26 In carefully selected women undergoing VBAC, a trial of low forceps or outlet forceps, or vacuum, may be attempted by a skilled obstetrician in an operating theater, where immediate recourse to CS can be undertaken, in case of any complication. This is discussed in detail, with recommendations for practice, in an upcoming paper on assisted vaginal birth from the FIGO Committee on Childbirth and PPH.
- **Facility requirements and intrapartum care during VBAC**
  28 VBAC should only be performed in facilities with resources for 24‐h emergency CS, blood bank, and NICU backup owing to associated maternal and neonatal risks.
  29 Supportive one‐to‐one midwifery/nursing care in labor and continuous electronic fetal monitoring are recommended, as fetal heart rate abnormality is the most common sign of uterine rupture.
  30 Epidural analgesia is not contraindicated during VBAC.
  31 The same standards and recommendations for evaluating labor progress in women without a previous CS apply to women undergoing VBAC.
  32 Routine uterine exploration after VBAC is not recommended in a hemodynamically stable patient with no abnormal vaginal bleeding.
  33 Surgical repair of asymptomatic uterine scar dehiscence suspected by uterine exploration after VBAC is not recommended.

## 9 CONCLUSION

VBAC can be an effective way to reduce the rising global CS rate. It can be performed with low absolute maternal and neonatal risks after one previous CS, in the absence of contraindications to VBAC and vaginal birth.

Maternal and neonatal outcomes of VBAC after one CS are comparable with planned repeat CS according to available current literature, and VBAC has the advantage of obviating the short‐ and long‐term risks of repeat CS.

In the presence of favorable factors, success rates of VBAC range from 60% to 80%, with a previous vaginal birth (particularly a VBAC) being the single best predictor of success.

Measures to safely increase VBAC attempt rates should be pursued globally, to stem the increasing tide of CS rates worldwide, and to ensure women have access to truly informed choice and decision‐making.

## AUTHOR CONTRIBUTIONS

E.R.B., D.R., A.E.U., N.D., and M.M. contributed to study conceptualization, planning, and design. E.R.B., D.R., and A.E.U. first coined the concept of the paper and its utmost value to FIGO. E.R.B., D.R., A.E.U., N.D., M.M., A.B.‐P., A.G., and C.B. contributed to the literature search, review, and evidence synthesis. E.R.B., D.R., and A.E.U. wrote the first manuscript draft. N.D., M.M., A.B.‐P., A.G., C.B., J.B.‐K., I.N., B.J., and A.W. reviewed, revised, and edited the first manuscript draft for sound intellectual, contemporary, and current best‐evidence content. A.W. coordinated the reviews, revisions, and edits while E.R.B. and A.E.U. implemented the reviews, revisions, and edits to produce a final manuscript. All authors read and approved the final manuscript.

## CONFLICT OF INTEREST STATEMENT

No conflicts to declare.

## Data Availability

Data sharing is not applicable to this article as no new data were created or analyzed in this study.

## References

[1] Ayres‐de‐Campos D , Simon A , Modi N , et al. European Association of Perinatal Medicine (EAPM) European Midwives Association (EMA) joint position statement: caesarean delivery rates at a country level should be in the 15–20% range. Eur J Obstet Gynecol Reprod Biol. 2024;294:76‐78. 38218162 10.1016/j.ejogrb.2024.01.005

[2] Visser GH , Ayres‐de‐Campos D , Barnea ER , et al. FIGO position paper: how to stop the caesarean section epidemic. Lancet. 2018;392(10155):1286‐1287. 30322563 10.1016/S0140-6736(18)32113-5

[3] Visser GHA , Ubom AE , Neji K , et al. FIGO opinion paper: drivers and solutions to the cesarean delivery epidemic with emphasis on the increasing rates in Africa and southeastern Europe. Int J Gynaecol Obstet. 2023;163(S2):5‐9. 37807592 10.1002/ijgo.15111

[4] Betran AP , Ye J , Moller AB , Souza JP , Zhang J . Trends and projections of caesarean section rates: global and regional estimates. BMJ Glob Health. 2021;6(6):e005671. 10.1136/bmjgh-2021-005671 PMC8208001 34130991

[5] Ramasauskaite D , Nassar A , Ubom AE , Nicholson W , FIGO Childbirth and Postpartum Hemorrhage Committee . FIGO good practice recommendations for cesarean delivery on maternal request: challenges for medical staff and families. Int J Gynaecol Obstet. 2023;163:10‐20. 37807587 10.1002/ijgo.15118

[6] Keag OE , Norman JE , Stock SJ . Long‐term risks and benefits associated with cesarean delivery for mother, baby, and subsequent pregnancies: systematic review and meta‐analysis. PLoS Med. 2018;15(1):e1002494. 29360829 10.1371/journal.pmed.1002494 PMC5779640

[7] Wang S , Hu Q , Liao H , Wang K , Yu H . Perinatal outcomes of pregnancy in women with scarred uteri. Int J Women's Health. 2023;15:1453‐1465. 37746587 10.2147/IJWH.S422187 PMC10517689

[8] Liu S , Liston RM , Joseph KS , Heaman M , Sauve R , Kramer MS . Maternal mortality and severe morbidity associated with low‐risk planned cesarean delivery versus planned vaginal delivery at term. CMAJ. 2007;176:455‐460. 17296957 10.1503/cmaj.060870 PMC1800583

[9] Silver RM , Landon MB , Rouse DJ , et al. Maternal morbidity associated with multiple repeat cesarean deliveries. Obstet Gynecol. 2006;107:1226‐1232. 16738145 10.1097/01.AOG.0000219750.79480.84

[10] Pineles BL , Buskmiller CM , Qureshey EJ , Stephens AJ , Sibai BM . Recent trends in term trial of labor after cesarean by number of prior cesarean deliveries. AJOG Glob Rep. 2023;3:100232. 37342471 10.1016/j.xagr.2023.100232 PMC10277578

[11] Boabang P , Bogesits‐Aufschneider R , Hug K . Vaginal delivery after two previous cesarean sections. Zentralbl Gynakol. 1999;121:449‐453. 10522379

[12] Lopian M , Perlman S , Cohen R , et al. TOLAC after 2 previous cesarean deliveries (TOLAC2). Why not? Am J Obstet Gynecol. 2023;228:S705‐S706.

[13] Shanks AL , Cahill AG . Delivery after prior cesarean: success rate and factors. Clin Perinatol. 2011;38:233‐245. 21645792 10.1016/j.clp.2011.03.011

[14] Bruno A , Allshouse A , Metz T . Trends in attempted and successful trial of labor after cesarean delivery in the United States from 2010 to 2020. Obstet Gynecol. 2023;141:173‐175. 36701618 10.1097/AOG.0000000000004998 PMC10477004

[15] Dy J , DeMeester S , Lipworth H , Barrett J . No. 382‐trial of labour after caesarean. J Obstet Gynaecol Can. 2019;41:992‐1011. 31227063 10.1016/j.jogc.2018.11.008

[16] Li WH , Yang MJ , Wang PH , et al. Vaginal birth after cesarean section: 10 years of experience in a tertiary medical center in Taiwan. Taiwan J Obstet Gynecol. 2016;55:394‐398. 27343322 10.1016/j.tjog.2016.04.016

[17] Lehmann S , Baghestan E , Børdahl PE , Irgens LM , Rasmussen S . Low risk pregnancies after a cesarean section: determinants of trial of labor and its failure. PLoS One. 2020;15(1):e0226894. 31929542 10.1371/journal.pone.0226894 PMC6957160

[18] Adebayo FO , Muhammad RB , Adewole N , Adesope AO . Trial of labour after caesarean section: a 5‐year review. Open J Obstet Gynecol. 2018;8:1121‐1129.

[19] Maroyi R , Naomi B , Moureau MK , et al. Factors associated with successful vaginal birth after a primary cesarean section in women with an optimal inter‐delivery interval. Int J Women's Health. 2021;13:903‐909. 34675688 10.2147/IJWH.S334269 PMC8502045

[20] Misgan E , Gedefaw A , Negash S , Asefa A . Validation of a vaginal after cesarean delivery prediction model in teaching hospitals of Addis Ababa university: a cross‐sectional study. Biomed Res Int. 2020;2020:1540460. 33015154 10.1155/2020/1540460 PMC7520680

[21] Boulvain M , Fraser WD , Brisson‐Carroll G , Faron G , Wollast E . Trial of labour after caesarean section in sub‐Saharan Africa: a meta‐analysis. Br J Obstet Gynaecol. 1997;104:1385‐1390. 9422017 10.1111/j.1471-0528.1997.tb11008.x

[22] Mascarello KC , Matijasevich A , Barros AJ , Santos IS , Zandonade E , Silveira MF . Repeat cesarean section in subsequent gestation of women from a birth cohort in Brazil. Reprod Health. 2017;14:1‐7. 28841832 10.1186/s12978-017-0356-8 PMC5572067

[23] Fitzpatrick KE , Kurinczuk JJ , Bhattacharya S , Quigley MA . Planned mode of delivery after previous cesarean section and short‐term maternal and perinatal outcomes: a population‐based record linkage cohort study in Scotland. PLoS Med. 2019;16:e1002913. 31550245 10.1371/journal.pmed.1002913 PMC6759152

[24] Lehmann S , Baghestan E , Børdahl PE , Irgens LM , Rasmussen S . Perinatal outcome in births after a previous cesarean section at high trial of labor rates. Acta Obstet Gynecol Scand. 2019;98:117‐126. 30192982 10.1111/aogs.13458

[25] Guise JM , Denman MA , Emeis C , et al. Vaginal birth after cesarean: new insights on maternal and neonatal outcomes. Obstet Gynecol. 2010;115:1267‐1278. 20502300 10.1097/AOG.0b013e3181df925f

[26] Landon MB , Hauth JC , Leveno KJ , et al. Maternal and perinatal outcomes associated with a trial of labor after prior cesarean delivery. N Engl J Med. 2004;351:2581‐2589. 15598960 10.1056/NEJMoa040405

[27] Al‐Zirqi I , Stray‐Pedersen B , Forsén L , Vangen S . Uterine rupture after previous caesarean section. BJOG. 2010;117:809‐820. 20236103 10.1111/j.1471-0528.2010.02533.x

[28] Tahseen S , Griffiths M . Vaginal birth after two caesarean sections (VBAC‐2)—a systematic review with meta‐analysis of success rate and adverse outcomes of VBAC‐2 versus VBAC‐1 and repeat (third) caesarean sections. BJOG. 2010;117:5‐19. 19781046 10.1111/j.1471-0528.2009.02351.x

[29] Macones GA , Cahill A , Pare E , et al. Obstetric outcomes in women with two prior cesarean deliveries: is vaginal birth after cesarean delivery a viable option? Am J Obstet Gynecol. 2005;192:1223‐1228. 15846208 10.1016/j.ajog.2004.12.082

[30] Cahill AG , Tuuli M , Odibo AO , Stamilio DM , Macones GA . Vaginal birth after caesarean for women with three or more prior cesareans: assessing safety and success. BJOG. 2010;117:422‐427. 20374579 10.1111/j.1471-0528.2010.02498.x

[31] Boatin AA , Cueto PD , Garba DL , et al. Trial of labour after caesarean section in sub‐Saharan Africa: a systematic review and meta‐analysis. Reprod Female Child Health. 2024;3:e104.

[32] Young CB , Liu S , Muraca GM , et al. Mode of delivery after a previous cesarean birth, and associated maternal and neonatal morbidity. CMAJ. 2018;190:E556‐E564. 29735533 10.1503/cmaj.170371 PMC5940456

[33] National Institutes of Health Consensus Development conference statement: vaginal birth after cesarean: new insights March 8–10, 2010. Obstet Gynecol. 2010;115:1279‐1295. 20502301 10.1097/AOG.0b013e3181e459e5

[34] Fitzpatrick KE , Quigley MA , Kurinczuk JJ . Planned mode of birth after previous cesarean section: a structured review of the evidence on the associated outcomes for women and their children in high‐income setting. Front Med. 2022;9:920647. 10.3389/fmed.2022.920647 PMC9486480 36148449

[35] Qiu L , Zhu J , Lu X . The safety of trial of labor after cesarean section (TOLAC) versus elective repeat cesarean section (ERCS): a systematic review and meta‐analysis. J Matern‐Fetal Neonatal Med. 2023;36:2214831. 37217450 10.1080/14767058.2023.2214831

[36] Chen X , Mi MY . The impact of a trial of labor after cesarean versus elective repeat cesarean delivery: a meta‐analysis. Medicine. 2024;103:e37156. 38363952 10.1097/MD.0000000000037156 PMC10869045

[37] Rezai S , Labine M , Gottimukkala S , et al. Trial of labor after cesarean (TOLAC) for vaginal birth after previous cesarean section (VBAC) versus repeat cesarean section: a review. Obstet Gynecol Int J. 2016;4:00135.

[38] Durnwald CP , Mercer BM . Vaginal birth after cesarean delivery: predicting success, risks of failure. J Matern Fetal Neonatal Med. 2004;15:388‐393. 15280110 10.1080/14767050410001724290

[39] Mekonnen BD , Asfaw AA . Predictors of successful vaginal birth after a cesarean section in Ethiopia: a systematic review and meta‐analysis. BMC Pregnancy Childbirth. 2023;23:65. 36703101 10.1186/s12884-023-05396-w PMC9878746

[40] Elkousy MA , Sammel M , Stevens E , Peipert JF , Macones G . The effect of birth weight on vaginal birth after cesarean delivery success rates. Am J Obstet Gynecol. 2003;188:824‐830. 12634665 10.1067/mob.2003.186

[41] Landon MB , Leindecker S , Spong CY , et al. The MFMU cesarean registry: factors affecting the success of trial of labor after previous cesarean delivery. Am J Obstet Gynecol. 2005;193:1016‐1023. 16157104 10.1016/j.ajog.2005.05.066

[42] Wu Y , Kataria Y , Wang Z , Ming WK , Ellervik C . Factors associated with successful vaginal birth after a cesarean section: a systematic review and meta‐analysis. BMC Pregnancy Childbirth. 2019;19:360. 31623587 10.1186/s12884-019-2517-y PMC6798397

[43] Weinstein D , Benshushan A , Tanos V , Zilberstein R , Rojansky N . Predictive score for vaginal birth after cesarean section. Am J Obstet Gynecol. 1996;174(1 Pt 1):192‐198. 8572005 10.1016/s0002-9378(96)70393-9

[44] Tessmer‐Tuck JA , El‐Nashar SA , Racek AR , Lohse CM , Famuyide AO , Wick MJ . Predicting vaginal birth after cesarean section: a cohort study. Gynecol Obstet Investig. 2014;77:121‐126. 24525697 10.1159/000357757

[45] Srinivas SK , Stamilio DM , Sammel MD , et al. Vaginal birth after caesarean delivery: does maternal age affect safety and success? Paediatr Perinat Epidemiol. 2007;21:114‐120. 17302640 10.1111/j.1365-3016.2007.00794.x

[46] Gonen R , Tamir A , Degani S , Ohel G . Variables associated with successful vaginal birth after one cesarean section: a proposed vaginal birth after cesarean section score. Am J Perinatol. 2004;21:447‐453. 15580540 10.1055/s-2004-835961

[47] Naef RW III , Ray MA , Chauhan SP , Roach H , Blake PG , Martin JN Jr . Trial of labor after cesarean delivery with a lower‐segment, vertical uterine incision: is it safe? Am J Obstet Gynecol. 1995;172:1666‐1673. 7778619 10.1016/0002-9378(95)91398-x

[48] Shipp TD , Zelop CM , Repke JT , Cohen A , Caughey AB , Lieberman E . Intrapartum uterine rupture and dehiscence in patients with prior lower uterine segment vertical and transverse incisions. Obstet Gynecol. 1999;94:735‐740. 10546720 10.1016/s0029-7844(99)00398-1

[49] Scott JR . Avoiding labor problems during vaginal birth after cesarean delivery. Clin Obstet Gynecol. 1997;40:533‐541. 9328733 10.1097/00003081-199709000-00011

[50] Greene RA , Fitzpatrick C , Turner MJ . What are the maternal implications of a classical caesarean section? J Obstet Gynaecol. 1998;18:345‐347. 15512105 10.1080/01443619867083

[51] Qayum K , Kar I , Sofi J , Panneerselvam H . Single‐ versus double‐layer uterine closure after cesarean section delivery: a systematic review and meta‐analysis. Cureus. 2021;13:e18405. 34729282 10.7759/cureus.18405 PMC8555931

[52] Bujold E , Gauthier RJ . Risk of uterine rupture associated with an interdelivery interval between 18 and 24 months. Obstet Gynecol. 2010;115:1003‐1006. 20410775 10.1097/AOG.0b013e3181d992fb

[53] Eden KB , McDonagh M , Denman MA , et al. New insights on vaginal birth after cesarean: can it be predicted? Obstet Gynecol. 2010;116:967‐981. 20859163 10.1097/AOG.0b013e3181f2de49

[54] Mercer BM , Gilbert S , Landon MB , et al. Labor outcomes with increasing number of prior vaginal births after cesarean delivery. Obstet Gynecol. 2008;111(2 Pt 1):285‐291. 18238964 10.1097/AOG.0b013e31816102b9

[55] ACOG practice bulletin no. 205: vaginal birth after cesarean delivery. Obstet Gynecol. 2019;133:e110‐e127. 30681543 10.1097/AOG.0000000000003078

[56] Flamm BL , Goings JR . Vaginal birth after cesarean section: is suspected fetal macrosomia a contraindication? Obstet Gynecol. 1989;74:694‐697. 2638575

[57] Chamagne M , Richard MB , Vallee A , et al. Trial of labour versus elective caesarean delivery for estimated large for gestational age foetuses after prior caesarean delivery: a multicenter retrospective study. BMC Pregnancy Childbirth. 2023;23:388. 37237350 10.1186/s12884-023-05688-1 PMC10214606

[58] Lessans N , Martonovits S , Rottenstreich M , et al. Trial of labor after cesarean in primiparous women with fetal macrosomia. Arch Gynecol Obstet. 2022;306:389‐396. 34709449 10.1007/s00404-021-06312-3

[59] Zelop CM , Shipp TD , Cohen A , Repke JT , Lieberman E . Trial of labor after 40 weeks' gestation in women with prior cesarean. Obstet Gynecol. 2001;97:391‐393. 11239643 10.1016/s0029-7844(00)01175-3

[60] Leung AS , Farmer RM , Leung EK , Medearis AL , Paul RH . Risk factors associated with uterine rupture during trial of labor after cesarean delivery: a case–control study. Am J Obstet Gynecol. 1993;168:1358‐1363. 8498412 10.1016/s0002-9378(11)90765-0

[61] Sentilhes L , Vayssière C , Beucher G , et al. Delivery for women with a previous cesarean: guidelines for clinical practice from the French College of Gynecologists and Obstetricians (CNGOF). Eur J Obstet Gynecol Reprod Biol. 2013;170:25‐32. 23810846 10.1016/j.ejogrb.2013.05.015

[62] Reif P , Brezinka C , Fischer T , et al. Labour and childbirth after previous caesarean section: recommendations of the Austrian Society of Obstetrics and Gynaecology (OEGGG). Geburtshilfe Frauenheilkd. 2016;76:1279‐1286. 28017971 10.1055/s-0042-118335 PMC5177557

[63] Jude G , Fain A , Raker C , et al. The association between trial of labor after cesarean in obese patients and adverse maternal outcomes. Arch Gynecol Obstet. 2024;309:2421‐2426. 37389641 10.1007/s00404-023-07113-6

[64] Yao R , Crimmins SD , Contag SA , Kopelman JN , Goetzinger KR . Adverse perinatal outcomes associated with trial of labor after cesarean section at term in pregnancies complicated by maternal obesity. J Matern‐Fetal Neonatal Med. 2019;32:1256‐1261. 29172787 10.1080/14767058.2017.1404023

[65] Juhasz G , Gyamfi C , Gyamfi P , Tocce K , Stone JL . Effect of body mass index and excessive weight gain on success of vaginal birth after cesarean delivery. Obstet Gynecol. 2005;106:741‐746. 16199630 10.1097/01.AOG.0000177972.32941.65

[66] Alani WY , Dayoub N . Factors influencing successful vaginal birth after cesarean delivery. Bahrain Med Bull. 2017;39:24‐28.

[67] Obeidat N , Meri ZB , Obeidat M , et al. Vaginal birth after caesarean section (VBAC) in women with spontaneous labour: predictors of success. J Obstet Gynaecol. 2013;33:474‐478. 23815200 10.3109/01443615.2013.782275

[68] Royal College of Obstetricians and Gynaecologists . Green‐top Guideline No. 45: Birth after Previous Caesarean Birth. RCOG; 2015.

[69] Horgan R , Hossain S , Fulginiti A , et al. Trial of labor after two cesarean sections: a retrospective case–control study. J Obstet Gynaecol Res. 2022;48:2528‐2533. 35793784 10.1111/jog.15351 PMC9796916

[70] Roberge S , Demers S , Berghella V , Chaillet N , Moore L , Bujold E . Impact of single‐ vs double‐layer closure on adverse outcomes and uterine scar defect: a systematic review and meta‐analysis. Am J Obstet Gynecol. 2014;211:453‐460. 24912096 10.1016/j.ajog.2014.06.014

[71] Bennich G , Rudnicki M , Wilken‐Jensen C , Lousen T , Lassen PD , Wøjdemann K . Impact of adding a second layer to a single unlocked closure of a cesarean uterine incision: randomized controlled trial. Ultrasound Obstet Gynecol. 2016;47:417‐422. 26489989 10.1002/uog.15792

[72] Di Spiezio Sardo A , Saccone G , McCurdy R , Bujold E , Bifulco G , Berghella V . Risk of cesarean scar defect following single‐vs double‐layer uterine closure: systematic review and meta‐analysis of randomized controlled trials. Ultrasound Obstet Gynecol. 2017;50:578‐583. 28070914 10.1002/uog.17401

[73] Nunes I , Nicholson W , Theron G , et al. FIGO good practice recommendations on surgical techniques to improve safety and reduce complications during cesarean delivery. Int J Gynecol Obstet. 2023;163:21‐33. 10.1002/ijgo.15117 37807585

[74] Rao J , Fan D , Ma H , et al. Is there an optimal inter‐delivery interval in women who underwent trial of labor after cesarean delivery (TOLAC)? Reprod Health. 2022;19:14. 35057818 10.1186/s12978-021-01319-0 PMC8772215

[75] Shipp TD , Zelop CM , Repke JT , Cohen A , Lieberman E . Interdelivery interval and risk of symptomatic uterine rupture. Obstet Gynecol. 2001;97:175‐177. 11165577 10.1016/s0029-7844(00)01129-7

[76] Huang WH , Nakashima DK , Rumney PJ , Keegan KA Jr , Chan K . Interdelivery interval and the success of vaginal birth after cesarean delivery. Obstet Gynecol. 2002;99:41‐44. 11777508 10.1016/s0029-7844(01)01652-0

[77] Kessous R , Sheiner E . Is there an association between short interval from previous cesarean section and adverse obstetric and perinatal outcome? J Matern Fetal Neonatal Med. 2013;26:1003‐1006. 23311913 10.3109/14767058.2013.765854

[78] Esposito MA , Menihan CA , Malee MP . Association of interpregnancy interval with uterine scar failure in labor: a case‐control study. Am J Obstet Gynecol. 2000;183:1180‐1183. 11084563 10.1067/mob.2000.109046

[79] Stamilio DM , DeFranco E , Pare E , et al. Short interpregnancy interval: risk of uterine rupture and complications of vaginal birth after cesarean delivery. Obstet Gynecol. 2007;110:1075‐1082. 17978122 10.1097/01.AOG.0000286759.49895.46

[80] Kok N , Wiersma IC , Opmeer BC , De Graaf IM , Mol BW , Pajkrt E . Sonographic measurement of lower uterine segment thickness to predict uterine rupture during a trial of labor in women with previous cesarean section: a meta‐analysis. Ultrasound Obstet Gynecol. 2013;42:132‐139. 23576473 10.1002/uog.12479

[81] Kement M , Kement CE , Kokanali MK , Doganay M . Prediction of uterine dehiscence via machine learning by using lower uterine segment thickness and clinical features. AJOG Glob Rep. 2022;2:100085. 36536838 10.1016/j.xagr.2022.100085 PMC9758335

[82] Jastrow N , Demers S , Chaillet N , et al. Lower uterine segment thickness to prevent uterine rupture and adverse perinatal outcomes: a multicenter prospective study. Am J Obstet Gynecol. 2016;215:604.e1‐604.e6. 10.1016/j.ajog.2016.06.018 27342045

[83] Shinar S , Agrawal S , Hasan H , Berger H . Trial of labor versus elective repeat cesarean delivery in twin pregnancies after a previous cesarean delivery—a systematic review and meta‐analysis. Birth. 2019;46:550‐559. 31124186 10.1111/birt.12434

[84] Kabiri D , Masarwy R , Schachter‐Safrai N , et al. Trial of labor after cesarean delivery in twin gestations: systematic review and meta‐analysis. Am J Obstet Gynecol. 2019;220:336‐347. 30465748 10.1016/j.ajog.2018.11.125

[85] Paul B , Möllmann CJ , Kielland‐Kaisen U , et al. Maternal and neonatal outcome after vaginal breech delivery at term after cesarean section—a prospective cohort study of the Frankfurt Breech at Term Cohort (FRABAT). Eur J Obstet Gynecol Reprod Biol. 2020;252:594‐598. 32507288 10.1016/j.ejogrb.2020.04.030

[86] Homafar M , Gerard J , Turrentine M . Vaginal delivery after external cephalic version in patients with a previous cesarean delivery: a systematic review and meta‐analysis. Obstet Gynecol. 2020;136:965‐971. 33030882 10.1097/AOG.0000000000004065

[87] Zhang N , Ward H . Safety and efficacy of external cephalic version after a previous caesarean delivery: a systematic review. Aust N Z J Obstet Gynaecol. 2021;61:650‐657. 34169515 10.1111/ajo.13399

[88] Zhang H , Liu H , Luo S , Gu W . Oxytocin use in trial of labor after cesarean and its relationship with risk of uterine rupture in women with one previous cesarean section: a meta‐analysis of observational studies. BMC Pregnancy Childbirth. 2021;21:11. 33407241 10.1186/s12884-020-03440-7 PMC7786988

[89] Lydon‐Rochelle M , Holt VL , Easterling TR , Martin DP . Risk of uterine rupture during labor among women with a prior cesarean delivery. N Engl J Med. 2001;345:3‐8. 11439945 10.1056/NEJM200107053450101

[90] West HM , Jozwiak M , Dodd JM . Methods of term labour induction for women with a previous caesarean section. Cochrane Database Syst Rev. 2017;6(6):CD009792. 28599068 10.1002/14651858.CD009792.pub3 PMC6481365

[91] Borovac‐Pinheiro A , Inversetti A , Di Simone N , et al. FIGO good practice recommendations for induced or spontaneous labor at term: prep‐for‐labor triage to minimize risks and maximize favorable outcomes. Int J Gynecol Obstet. 2023;163(suppl. 2):51‐56. 10.1002/ijgo.15114 37807591

[92] World Health Organization . WHO Recommendations for Induction of Labour. WHO; 2011. 23586118

[93] Schmitz T , Pourcelot AG , Moutafoff C , Biran V , Sibony O , Oury JF . Cervical ripening with low‐dose prostaglandins in planned vaginal birth after cesarean. PLoS One. 2013;8:e80903. 24260505 10.1371/journal.pone.0080903 PMC3834249

[94] Haas J , Barzilay E , Chayen B , et al. Safety of low‐dose prostaglandin E2 induction in grandmultiparous women with previous cesarean delivery. J Matern Fetal Neonatal Med. 2014;27:445‐448. 23841832 10.3109/14767058.2013.818651

[95] Stock SJ , Ferguson E , Duffy A , Ford I , Chalmers J , Norman JE . Outcomes of induction of labour in women with previous caesarean delivery: a retrospective cohort study using a population database. PLoS One. 2013;8:e60404. 23565242 10.1371/journal.pone.0060404 PMC3615029

[96] Palatnik A , Grobman WA . Induction of labor versus expectant management for women with a prior cesarean delivery. Am J Obstet Gynecol. 2015;212:358.e1‐358.e6. 10.1016/j.ajog.2015.01.026 25725658

[97] Krizman E , Grzebielski P , Antony KM , et al. Operative vaginal delivery is a safe option in women undergoing a trial of labor after cesarean. AJP Rep. 2019;9:e190‐e194. 31218115 10.1055/s-0039-1692482 PMC6581533

[98] Son M , Roy A , Grobman WA . Attempted operative vaginal delivery vs repeat cesarean in the second stage among women undergoing a trial of labor after cesarean delivery. Am J Obstet Gynecol. 2017;216:407.e1‐407.e5. 10.1016/j.ajog.2017.01.013 28153660

[99] Barnea ER , Inversetti A , Di Simone N , et al. FIGO good practice recommendations for cesarean delivery: prep‐for‐labor triage to minimize risks and maximize favorable outcomes. Int J Gynecol Obstet. 2023;163(suppl. 2):57‐67. 10.1002/ijgo.15115 37807590

[100] Black N , Henderson I , Al Wattar BH , Quenby S . Predictive models for estimating the probability of successful vaginal birth after cesarean delivery: a systematic review. Obstet Gynecol. 2022;140:821‐841. 36201785 10.1097/AOG.0000000000004940

[101] Barnea ER , Muller M , Di Simone N , et al. Prep‐for‐labor: overview of FIGO's labor and delivery triage bundles of care to optimize maternal and newborn outcomes. Int J Gynecol Obstet. 2023;163(suppl. 2):34‐39. 10.1002/ijgo.15112 37807589

[102] Grisaru‐Granovsky S , Bas‐Lando M , Drukker L , et al. Epidural analgesia at trial of labor after cesarean (TOLAC): a significant adjunct to successful vaginal birth after cesarean (VBAC). J Perinat Med. 2018;46:261‐269. 28622143 10.1515/jpm-2016-0382

[103] Uppington J . Epidural analgesia and previous caesarean section. Anaesthesia. 1983;38:336‐341. 6846763 10.1111/j.1365-2044.1983.tb10456.x

[104] Sun J , Yan X , Yuan A , et al. Effect of epidural analgesia in trial of labor after cesarean on maternal and neonatal outcomes in China: a multicenter, prospective cohort study. BMC Pregnancy Childbirth. 2019;19:498. 31842795 10.1186/s12884-019-2648-1 PMC6916071

[105] Eshkoli T , Jacobs M , Saban A , et al. Impact of epidural analgesia on outcomes of vaginal birth after cesarean delivery. Arch Gynecol Obstet. 2025;312:131‐137. 39884975 10.1007/s00404-025-07959-y PMC12176991

[106] Graseck AS , Odibo AO , Tuuli M , Roehl KA , Macones GA , Cahill AG . Normal first stage of labor in women undergoing trial of labor after cesarean delivery. Obstet Gynecol. 2012;119:732‐736. 22433336 10.1097/AOG.0b013e31824c096c

[107] Shalev‐Ram H , Miller N , David L , Issakov G , Weinberger H , Biron‐Shental T . Spontaneous labor patterns among women attempting vaginal birth after cesarean delivery. J Matern Fetal Neonatal Med. 2022;35:9325‐9330. 35098866 10.1080/14767058.2022.2031964

